# Metabolic Risk Factors Are Associated With Weight Status Change Over Four Years in Children Aged 4–6 Years With Obesity

**DOI:** 10.1111/apa.70633

**Published:** 2026-06-05

**Authors:** Markus Brissman, Anna Ek, Stella Wennborg, Karin Nordin, Karin Eli, Annika Janson, Paulina Nowicka

**Affiliations:** ^1^ Karolinska Institutet, Department of Clinical Science, Intervention and Technology (CLINTEC), Division of Pediatrics Stockholm Sweden; ^2^ Allied Health Professionals Function, Occupational Therapy & Physiotherapy Karolinska University Hospital Stockholm Sweden; ^3^ National Childhood Obesity Centre Karolinska University Hospital Stockholm Sweden; ^4^ Warwick Medical School University of Warwick Coventry UK; ^5^ Department of Women's and Children's Health Karolinska Institutet Stockholm Sweden; ^6^ Department of Food Studies, Nutrition and Dietetics Uppsala University Uppsala Sweden

**Keywords:** childhood obesity, cholesterol, metabolic risk, parent‐focused treatment, triglycerides

## Abstract

**Aim:**

It is unclear how early childhood obesity treatment affects metabolic risk. This study assessed long‐term metabolic health in children with obesity aged 4–6 years and examined associations with weight status.

**Methods:**

This prospective cohort study pooled data from the Sweden‐based More and Less randomized controlled trial, which compared a parent support program with standard care in 2012–2016. Children met the International Obesity Task Force (IOTF) criteria for obesity. Anthropometrics and metabolic risk factors were collected at baseline, 12 and 48 months. Associations between metabolic markers and weight status were examined using linear mixed models.

**Results:**

Data from 106/177 (60%) children, mean age at baseline 5.3 years, were analysed. At baseline, 33/90 (36.6%) had elevated metabolic risk markers. At 48 months, one unit of body mass index standard deviation score (BMI SDS) was associated with higher glycosylated haemoglobin (HbA1c IFCC) (0.99 mmol/mol), higher fasting insulin (2.72 mIU/L) and lower high‐density lipoprotein (HDL) (−0.16 mmol/L or −6.2 mg/dL). A BMI SDS reduction ≥ 0.25 was associated with lower HbA1c and triglycerides at 12 months, and a reduction ≥ 0.5 with lower total cholesterol at 48 months.

**Conclusion:**

Improved weight status may reverse metabolic disturbances present in early childhood.

## Summary

- Little is known about how obesity treatment in early childhood influences metabolic health in children aged 4–6 years.
- In this four‐year follow‐up of 106 children with obesity, aged 4–6 years, one third had elevated metabolic risk markers at baseline; however, reductions in weight status following treatment were associated with decreased metabolic risk over time.
- Early childhood obesity treatment may reverse metabolic disturbances by reducing weight status.

## Graphical abstract

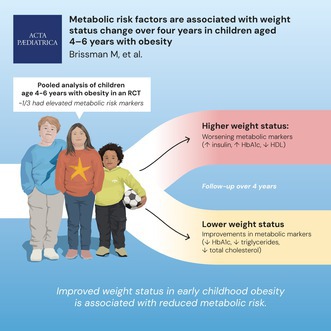

**Abbreviations**
%CDC95: the BMI of a child expressed in relation to the 95th percentile on the CDC growth chart
%IOTF25: the BMI of a child expressed in relation to the IOTF‐BMI 25 cutoff
ALT: alanine transaminase
AST: aspartate aminotransferase
BMI: body mass index
CDC: Centers for Disease Control and Prevention
HbA1c: glycosylated haemoglobin
HDL: high‐density lipoprotein
hsCRP: high‐sensitivity C‐reactive protein
IFCC: International Federation of Clinical Chemistry and Laboratory Medicine
IOTF: International Obesity Task Force
IU: international units
LDL: low‐density lipoprotein
SDS: standard deviation score
TC: total cholesterol

## 1 Introduction

Obesity in early childhood is associated with impaired metabolic health and is linked to a cumulative increased risk of adverse outcomes in adulthood [1] including impaired glucose tolerance and type 2 diabetes, cardiovascular disease, and non‐alcoholic fatty liver disease [2, 3]. As there is no established definition of the metabolic syndrome for children below ten years of age, the childhood obesity literature has referred to metabolic risk factors rather than the metabolic syndrome [4]. However, most studies on metabolic risk among children with obesity, including a national Swedish cohort study published in 2025 [5], have focused on children aged six years and older.

Low‐grade inflammation has been reported in children with obesity as young as two years old [6], suggesting that pathological processes associated with obesity are initiated early. Studies involving children with obesity aged 6–14 years have shown that a reduction in the degree of obesity may ameliorate metabolic risk factors, implying a reversal of the adverse effects associated with obesity [7, 8, 9, 10, 11]. However, data have been scarce, especially in early childhood, with limited knowledge on how obesity treatment may affect the metabolic profiles of children under the age of six years [4, 12, 13, 14].

To date, only two obesity treatment programs for preschool‐aged children, aged six years and younger, have been evaluated in randomized controlled trials [15]. One is a US‐based program for children aged 2–5 years [16]. The other is the More and Less, an evidence‐based parenting‐skills training program that outperformed standard care after 12 months by improving children's weight status [17], with outcomes extending to 48 months post baseline [18]. Alongside the primary weight‐related outcomes, metabolic risk factors were assessed as part of the More and Less trial.

The aim of this study was to assess long‐term metabolic health in children with obesity, aged 4–6 years, and examine associations between changes in child weight status and metabolic risk markers, using data from the More and Less study.

## 2 Patients and Methods

This study analysed pooled data of metabolic markers from the More and Less study, a parallel, open label randomized controlled trial comparing a parent support program with standard treatment of obesity in children of preschool age. The More and Less study took place in 2012–2016, in Stockholm, Sweden. The protocol for the More and Less study has been described in detail [19]. This secondary data analysis was performed in 2024–2026.

### 2.1 Participants

In total, 336 families were screened for eligibility for the More and Less study. Of these, 177 families were randomized 2:1:1 to standard treatment (*n* = 87), parent group non‐booster (*n* = 43) or parent group booster (*n* = 44). Both parent groups participated in the More and Less program, consisting of ten weekly 90‐min sessions. In the parent group booster, participating families also received booster sessions: a maximum of seven 30‐min phone calls, every four to six weeks for nine months after the parent program had ended. For this study, which assesses the long‐term metabolic health of children with obesity and its association with changes in child weight status, all three groups were pooled for analysis regardless of randomization.

### 2.2 Anthropometrics and Calculations

Anthropometrics were measured by the research team or by child health care professionals at the participating outpatient clinics, at baseline, 12 and 48 months in accordance with the study protocol [20]. The child's height was measured in centimetres using a fixed stadiometer and rounded to the nearest 0.1 cm. The child's weight was measured in kilograms rounded to the nearest 0.1 kg with the child in light clothing. Body mass index (BMI), kg/m^2^, was calculated, and BMI standard deviation scores (BMI SDS) were derived using the international age‐specific and sex‐specific references by the International Obesity Task Force (IOTF) [20]. Participants were categorized based on BMI SDS reduction of ≥ 0.25 units and ≥ 0.5 units, respectively. These cutoffs match previous studies and are associated with improvements in metabolic health in children and adolescents [10, 21, 22]. Additionally, %IOTF25 [23, 24] was calculated to illustrate the child's BMI in relation to the IOTF‐BMI 25 cutoff for overweight, adjusted for sex and age, used in Sweden. This metric resembles the Centers for Disease Control and Prevention (CDC) index %CDC95 [25], which expresses BMI relative to the 95th percentile. The %IOTF25 was calculated by dividing the child's BMI by the IOTF‐BMI 25 cutoff and multiplying by 100. A score of 100 equals the cutoff for overweight. The child's waist circumference was measured between the lower ribcage and iliac crest using nonextensile tape, with the result rounded to the nearest 0.1 cm. All measurements were taken using calibrated instruments and repeated three times, of which a mean value was derived.

### 2.3 Blood Chemistry

Fasting blood samples were taken at baseline and at the 12‐month and 48‐month follow‐up. Testing was performed in accredited laboratories and results were obtained from the children's electronic medical records. The assessed metabolic markers were high sensitivity C‐reactive protein (hsCRP), glucose, glycosylated haemoglobin (HbA1c), insulin, total cholesterol (TC), low‐density lipoprotein (LDL), high‐density lipoprotein (HDL), triglycerides, and the liver enzymes alanine transaminase (ALT) and aspartate aminotransferase (AST). Values above 10 mg/L for hsCRP were not included in the analyses to distinguish low‐grade from acute inflammation [26]. Reference values for HbA1c, LDL, HDL, triglycerides, ALT and AST were obtained from Karolinska University Laboratory [27] (Data S1). In addition, liver‐transaminases were considered elevated if they were ≥ 2 times the upper reference interval, a proxy indicating non‐alcoholic fatty liver disease [28]. For insulin, age‐ and sex‐dependent 95th percentile cutoffs were adopted from the Identification and prevention of Dietary‐ and lifestyle‐induced health EFfects In Children and infantS (IDEFICS) consortium [29].

### 2.4 Sociodemographics

Baseline questionnaires were used to collect sociodemographic information. Children were identified as having migrant background if at least one parent and both grandparents were born outside Sweden. Parents were described as university‐educated if at least one parent had a university degree.

### 2.5 Statistics

Descriptive statistics with means and standard deviations (SD) or frequencies and percentages are reported unless otherwise specified. A linear mixed model with restricted maximum likelihood and unstructured covariance was used for the analysis of the main effects: changes in metabolic markers over time, adjusted for BMI SDS, age, sex, parental education level, and migrant background. Means with 95% confidence intervals (95% CI) were reported. Dichotomous analysis of risk marker prevalence was performed using a generalized linear mixed model. Associations between metabolic markers and cutoffs of BMI SDS > 0.25 and > 0.5 were analysed separately at 12 and 48 months. All analyses were two‐tailed and a *p*‐value < 0.05 was considered statistically significant. SPSS statistics version 28 (IBM Corp, New York, USA) was used.

Participation was defined as having metabolic marker values at baseline and at least one time‐point after baseline.

### 2.6 Ethics

The study was approved by the Regional Ethical Board in Stockholm (ID: 2011/1329–31/4) with amendments (2012/1104–32; 2012/2005–32; 2013/486–32). The More and Less study protocol was registered with the clinical trials registry clinicaltrials.gov (ID: NCT01792531). Parental consent for this secondary analysis was not required as consent was obtained as part of the original More and Less trial.

## 3 Results

### 3.1 Characteristics

**FIGURE 1:**
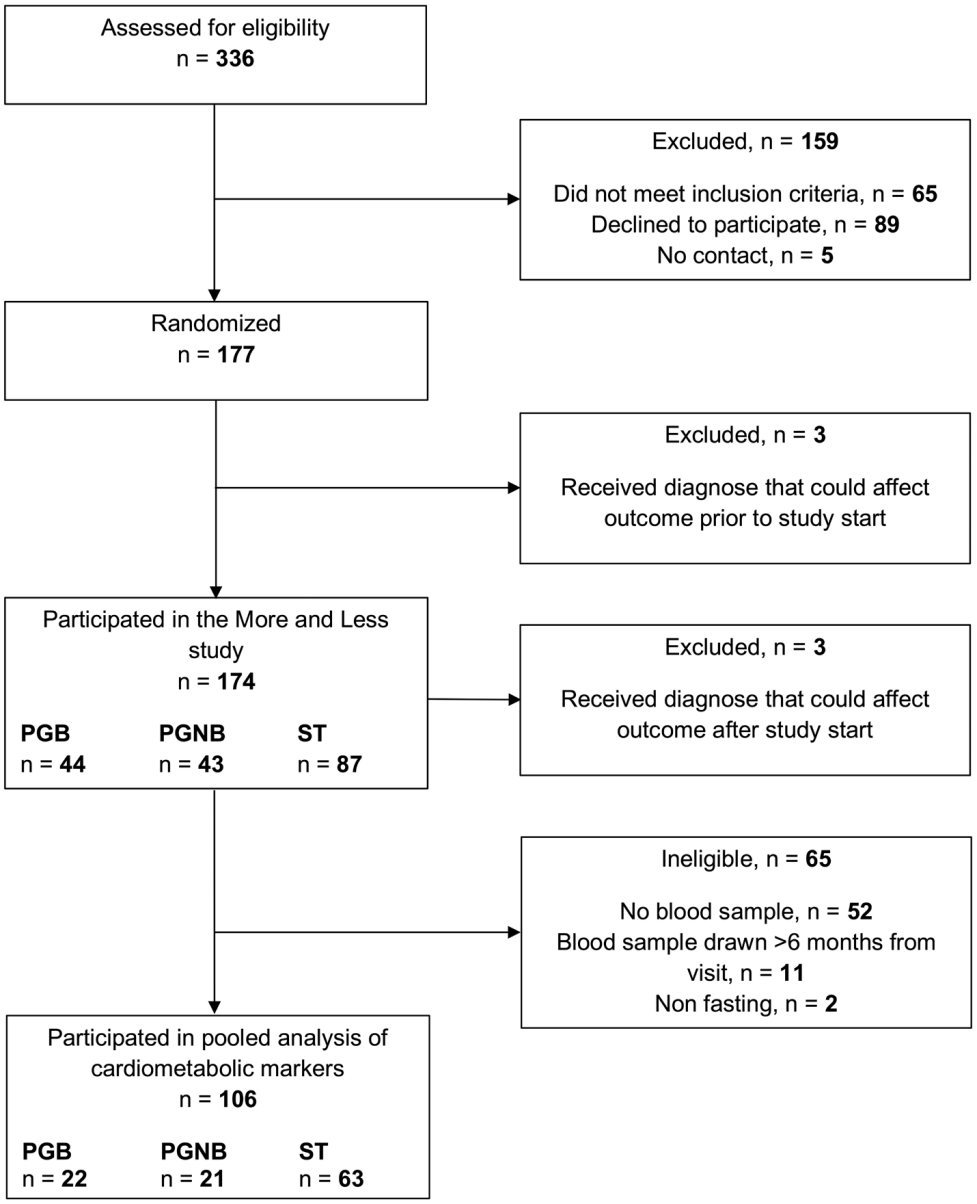
Study participant flowchart. PGB, Parent Group Booster; PGNB, Parent Group Non‐Booster; ST, Standard Treatment.

The total sample of 106 children included 62 girls and 44 boys. At baseline, the mean age was 5.3 (0.7) years and BMI SDS was 3.0 (0.6) (Table 1). From the original More and Less study of 177 children, six were excluded after receiving diagnoses that could affect physical or metabolic development: one each with, leukaemia, type 1 diabetes, Prader–Willi syndrome, and Klinefelter syndrome, and two with autism. A further 65 were ineligible: in 52 cases, no blood samples were collected, in 11 cases, blood samples were drawn more than six months before or after anthropometric measurements were taken, and in two cases, non‐fasting blood samples were collected (Figure 1). No significant difference in weight, BMI, BMI SDS or age was found between included and excluded/ineligible children at baseline, 12 or 48 months.

**TABLE 1:** Baseline characteristics.

	*n*	Mean (SD)
Age	106	5.3 (0.7)
Sex female, *n* (%)	106	62 (58.5)
BMI‐SDS^a^	106	3.0 (0.6)
%IOTF25^b^	106	123.5 (10.4)
BMI	106	21.4 (1.8)
Waist circumference (cm)	93	66.9 (6.0)
Height (cm)	106	117.5 (6.9)
Weight (kg)	106	29.7 (4.7)
Parents' university degree^c^, *n* (%)	101	53 (52.5)
Migrant background^d^, *n* (%)	101	69 (68.3)

^a^ BMI‐SDS: Body Mass Index Standard Deviation Score according to International Obesity Task Force (IOTF).

^b^ The distance in percentage above the IOTF cut‐off for overweight.

^c^ Parents' university degree: At least one parent with a university degree.

^d^ Migrant background: at least one parent and both grandparents being born outside Sweden.

### 3.2 Metabolic Profile

At baseline, most children had metabolic risk markers within reference ranges (Table 2). Nevertheless, 33/90 (36.6%) of children had one or more elevated risk markers. At 12 months, 16/44 (36.4%) of the children had at least one or more elevated risk markers. By 48 months, elevated risk markers were found in most children, with 21/33 (63.6%) having one or more elevated values. Comprehensive data for all included risk markers is presented in Table 2.

**TABLE 2:** Blood chemistry, baseline, 12‐ and 48 months with mean, range and % above threshold of the normal range.

	Baseline				12‐month				48‐month			
	*n*	Mean (SD)	range	% above threshold	*n*	Mean (SD)	range	% above threshold	*n*	Mean (SD)	range	% above threshold
hsCRP [mg/L]	68	1.6 (1.7)	0.2–9.0	14.7	33	1.5 (1.5)	0.2–6.6	18.2	22	1.8 (1.4)	0.0–6.0	22.7
Insulin [mIU/L]	77	6.9 (3.8)	1.4–21.0	18.2^a^	41	7.6 (6.2)	2.2–38	22.0^a^	30	20.8 (17.3)	2–80	56.6^a^
Glucose [mmol/L]	86	4.9 (0.4)	4.1–5.9	—	44	4.9 (0.4)	3.7–6.0	—	31	5.2 (0.3)	4.4–5.9	—
HbA1c [mmol/mol]	84	32.7 (3.0)	24–41	—	44	32.3 (2.5)	26–38	—	31	34.3 (2.6)	28–40	—
TC [mmol/L]	88	4.1 (0.7)	0.6–5.9	—	43	4.1 (0.7)	2.9–6.0	2.3	29	4.3 (0.7)	2.9–5.6	—
LDL [mmol/L]	87	2.5 (0.5)	1.2–3.9	—	43	2.4 (0.6)	1.5–4.0	2.3	30	2.5 (0.6)	1.2–4.0	3.3
HDL [mmol/L]	89	1.4 (0.3)	0.7–2.3	—	43	1.5 (0.3)	1.0–2.2	—	30	1.3 (0.3)	0.8–1.9	—
TG [mmol/L]	90	0.8 (1.0)	0.2–2.2	—	43	0.6 (0.4)	0.3–2.6	—	31	1.1 (0.7)	0.4–3.2	6.5
ALT [mikrokat/L]	90	0.4 (0.1)	0.2–0.7	11.1	44	0.4 (0.2)	0.2–1.5	15.9	32	0.5 (0.4)	0.2–1.6	28.1
AST [mikrokat/L]	83	0.6 (0.1)	0.3–1.1	4.8	42	0.6 (0.1)	0.4–1.0	4.8	31	0.5 (0.2)	0.3–1.1	9.7

Abbreviations: ALT, alanine transaminase; AST, aspartate aminotransferase; HbA1c, glycosylated haemoglobin; HDL, high‐density lipoprotein; hsCRP, C‐reactive protein; IU, international units; LDL, low‐density lipoprotein; TC, total cholesterol; TG, triglycerides.

^a^ Idefics above 95th percentile.

The prevalence of elevated insulin increased markedly, from 18.2% at baseline to 56.6% after 48 months (*p* < 0.001). An increase in the proportion of children with elevated levels of ALT was seen over time, from 11.1% at baseline to 28.1% after 48 months, but this increase was not significant. No child had values ≥ 2 times upper reference interval for ALT or AST. For LDL, 2.3% of the children had elevated values at baseline and 3.3% at 48 months, which was not significant. For TC, 2.3% of children had elevated values at 12 months and 0% at 48 months, which was not significant. No child had elevated glucose, HbA1c or low HDL at any measure point.

### 3.3 Metabolic Risk Markers Over Time

A positive association was found between several metabolic risk markers and increasing BMI SDS (per unit) over time (95% CI): hsCRP 0.72 mg/L (95% CI 0.33 to 1.12, *p* < 0.001), HbA1c 0.99 mmol/mol (95% CI 0.26 to 1.72, *p* = 0.009), fasting insulin 2.34 mIU/L (95% CI 1.00 to 3.68, *p* < 0.001), fasting glucose 0.11 mmol/L (95% CI 0.004 to 0.22, *p* = 0.043) ALT 0.04 microkat/L (95% CI 0.002 to 0.075, *p* = 0.041) and triglycerides 0.135 mmol/L (95% CI 0.03 to 0.24 *p* = 0.010). A negative association was found between HDL and BMI SDS 0.16 mmol/L (−0.24 to −0.08, *p* < 0.001). Associations were adjusted for gender, age, migrant background and parental university degree. No significant association was found between BMI SDS and TC, LDL or AST.

A similar pattern of positive associations between weight status and metabolic risk markers was found per unit (%) IOTF25% over time (95% CI): hsCRP 0.04 mg/L (95% CI 0.02 to 0.06, *p* < 0.001), HbA1c 0.05 mmol/L (95% CI 0.02 to 0.09, *p* = 0.003), fasting insulin 0.17 mIU/L (95% CI 0.9 to 2.5, *p* < 0.001), ALT 0.002 microkat/L (95% CI 0.000153 to 0.004, *p* = 0.038), triglycerides 0.009 mmol/L (95% CI 0.002 to 0.016, *p* = 0.008). A negative association was identified between IOTF25% and HDL −0.09 mmol/L (−0.05 to −0.12, *p* < 0.001). Associations were adjusted for sex, age, migrant background and parental university degree. No significant association was found between IOTF25% and fasting glucose, TC, LDL or AST.

### 3.4 Metabolic Profile and Change in BMI SDS

Overall, 61/84 (72.6%) of participants experienced a reduction of BMI SDS from baseline to 48 months. A BMI SDS reduction of ≥ 0.5 was found in 31/84 (36.9%) of the participants and 16/84 (19.1%) reduced their BMI SDS by ≥ 0.25. A reduction of < 0.25 to < 0.0 was found in 14/84 (16.6%) and 23/84 (27.4%) had BMI SDS equal to or higher than baseline.

A reduction of BMI SDS by ≥ 0.25 at 12 months was associated with lower levels of HbA1c 1.37 mmol/mol (95% CI −0.28 to −2.46, *p* = 0.015) and triglycerides −0.25 (95% CI −0.5 to −0.03, *p* = 0.02). At 48 months, a reduction of BMI SDS by ≥ 0.5 was associated with lower levels of TC 0.3 mmol/L (95% CI −0.57 to −0.03, *p* = 0.03). For other metabolic markers (hsCRP, glucose, insulin, LDL, HDL, ALT and AST), results were not significant.

**FIGURE 2:**
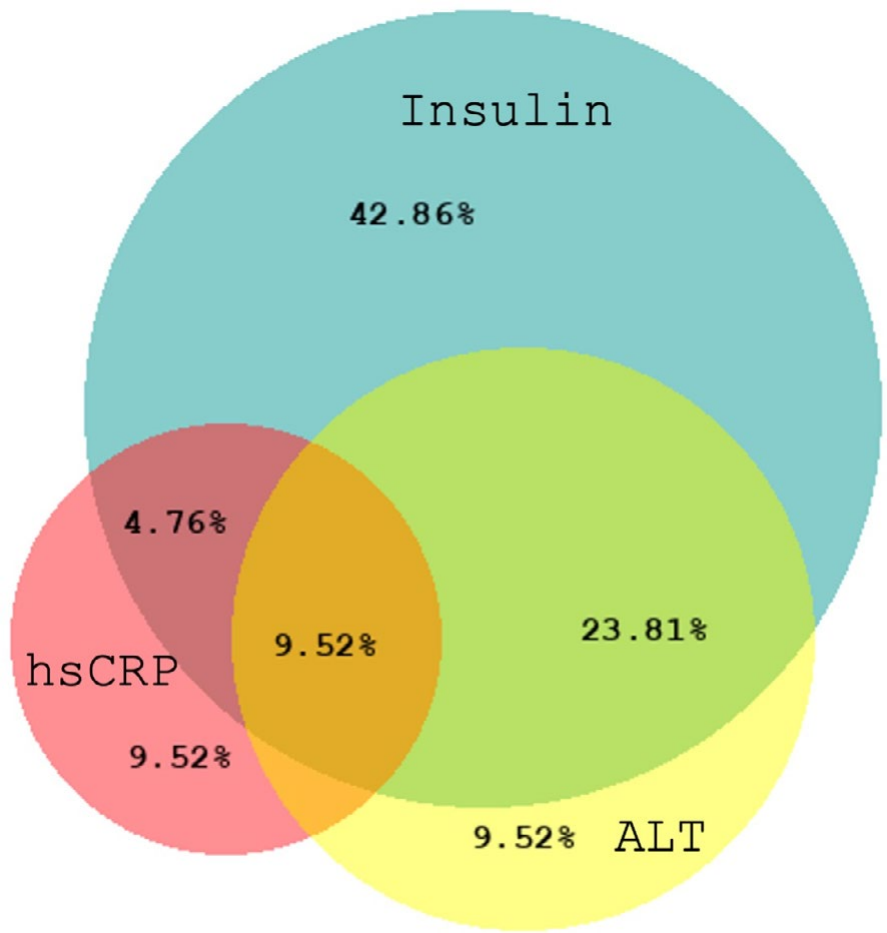
At 48 months there was an overlap between the most common elevated metabolic risk markers, fasting insulin, alanine transaminase (ALT) and high sensitivity C‐reactive protein (hsCRP).

At 48 months, there was an overlap between the most commonly elevated metabolic risk markers: hsCRP, insulin and ALT (Figure 2). Of the 17 children with elevated insulin, 13 had other elevated metabolic risk markers. Of the five children with elevated hsCRP, three (60%) had elevated insulin. The child with elevated LDL had elevated insulin, both children with elevated triglycerides had elevated insulin, and seven of the nine children with elevated ALT had elevated insulin (77.8%).

## 4 Discussion

In this pooled analysis of participants in a randomized controlled trial, we found a marked prevalence of metabolic risk factors in young children with obesity, aged 4–6 years at baseline. Over a period of four years, increased BMI SDS was associated with worsening in some, but not all, metabolic markers. A reduction of ≥ 0.25 and ≥ 0.5 BMI SDS had a limited but beneficial effect on some metabolic markers. Overall, the prevalence of elevated metabolic risk markers increased over time. Indications of impaired fasting insulin were found in 18.2% of children at baseline and in 56.6% after 48 months. There was also an increased prevalence of elevated hsCRP and ALT levels over the study period. These results highlight that the negative health consequences of obesity begin to develop in early childhood.

The high prevalence of inflammatory markers was comparable to findings from a study [6] which showed an association between elevated hsCRP and obesity among young children. This earlier study found that higher levels of hsCRP predict a larger increase in BMI SDS and obesity incidence in children [6]. The current study, however, found no such association, conveying the need for further longitudinal studies of inflammatory markers in early childhood.

BMI SDS was associated with both insulin and HbA1c over time, confirming the known association between obesity, impaired glucose tolerance, and insulin resistance in children [7, 8, 10, 30]. However, this study clearly indicates that this association starts at age 4–6 years. Importantly we found that a reduction of BMI SDS by ≥ 0.25 had a favourable effect on glucose homeostasis (HbA1c) after 12 months. This finding echoed similar results from Reinehr et al.'s study with children aged 5–17 years [21]. Together, these studies show a reversal of the adverse effects of obesity on glucose homeostasis with successful treatment.

The inverse relationship between BMI SDS and HDL over time showed that obesity is associated with an adverse lipid profile already at this young age. In parallel with HbA1c, a reduction of BMI SDS by ≥ 0.25 units had beneficial effects on triglyceride levels after 12 months. These results supported the conclusion by Reinehr et al. [21] that this BMI SDS reduction is sufficient for improving metabolic health in children, and further showed that this cutoff is also significant for children enrolled in obesity treatment aged 4–6 years. At the 48‐month follow‐up, a reduction of BMI SDS by ≥ 0.5 was associated with lower values of TC. This cutoff has previously been shown to improve the metabolic profile of older children in a shorter‐term follow‐up [9, 22]. Our findings supported this finding among young children and further suggested that long term reduction of BMI SDS has metabolic benefits.

Elevated liver enzymes were the most common deviation from reference intervals at baseline. Notably, no values were ≥ 2 times age‐appropriate upper reference limits. While such thresholds are sometimes used for risk stratification, our findings should not be interpreted as indicative of non‐alcoholic fatty liver disease, but rather as possible early or subclinical liver dysfunction [28]. Previous cross‐sectional studies have demonstrated a higher prevalence of elevated liver enzymes among children aged five years and older and adolescents with obesity [31]. However, results regarding the impact of the degree of obesity have been contradictory [31, 32]. The results of our study did not support an association between higher BMI SDS and prevalence of elevated liver enzymes.

### 4.1 Strengths and Limitations

This study had several strengths. To the best of our knowledge, it was the first study to analyse the metabolic effects of obesity in young children. It was also the first to offer a longitudinal analysis of metabolic health data over 48 months. Furthermore, the diverse study population, which included a range of educational and migrant backgrounds, increased the generalizability of these results. The study's main limitation was that the number of participants was not tailored for the analyses of these secondary outcomes as power was calculated for the primary outcome of the More and Less study, BMI SDS [18]. In addition, due to the circumstances caused by the COVID‐19 pandemic, we had to include blood test data obtained up to six months before or after the anthropometric measures. The study could have been strengthened by collecting data on changes in the participants' dietary intake, as these may have affected risk markers. In addition, data on infections just before or during blood sampling would have allowed us to place the hsCRP results in wider context. However, since much of the sampling occurred during COVID‐19 restrictions, we assumed that children were not symptomatic of fever or respiratory infections when blood samples were taken. Furthermore, for hsCRP, we chose to omit values > 10 mg/L as suggested by others [26] to distinguish between low‐grade and acute inflammation. Finally, for a large number of participants, blood samples were missing at the 48‐month follow‐up. We carefully performed missing case analysis to investigate possible bias and found no differences between completers and non‐completers. The increased prevalence of higher fasting insulin at the 48‐month follow‐up should be interpreted with caution, considering the normally occurring insulin resistance in the early stages of puberty, but no child was older than 11.5 years at the 48‐month follow‐up.

## 5 Conclusion

This study found early signs of metabolic risk factors associated with obesity among children aged 4–6 years. The association between high BMI SDS and impaired glucose homeostasis, along with an adverse lipid profile, mirrored associations previously seen in older children. The findings demonstrated that obesity among young children may lead to serious health consequences later in life. However, the findings also showed that a reduction of BMI SDS was associated with a reversal of metabolic disturbances. This suggests that initiating childhood obesity treatment early may significantly reduce metabolic risk.

## Author Contributions

**Markus Brissman:** conceptualization, writing – original draft, formal analysis, supervision, data curation, methodology. **Anna Ek:** conceptualization, funding acquisition, methodology, writing – review and editing, data curation. **Stella Wennborg:** writing – original draft, formal analysis, conceptualization. **Karin Nordin:** writing – review and editing, data curation, resources. **Karin Eli:** writing – review and editing. **Annika Janson:** writing – review and editing, methodology. **Paulina Nowicka:** conceptualization, funding acquisition, writing – review and editing, methodology, project administration, supervision, data curation, resources.

## Funding

This work was supported by Stiftelsen Frimurare Barnhuset i Stockholm. Center for Innovative Medicine, SLL20190383.

## Conflicts of Interest

The authors declare no conflicts of interest.

## Supporting information


**Data S1:** Attachment 1 Paediatric reference ranges for metabolic risk factors R2.

## Data Availability

The data that support the findings of this study are available from the corresponding author upon reasonable request.
