# Supplementary material for: Metabolic Risk Factors Are Associated With Weight Status Change Over Four Years in Children Aged 4–6 Years With Obesity
Source: Acta Paediatr. 2026 Jun 5;115(9):1891–8. doi: 10.1111/apa.70633 (PMC13460735; doi:10.1111/apa.70633)
Supplement: Supplementary file 1 [file APA-115-1891-s001.docx]

**Pediatric reference ranges for metabolic risk factors associated with obesity**

| Marker | Reference range |
| --- | --- |
|  |  |
| hsCRP [mg/L] | <3 |
| Glucose [mmol/L] | 4.2 – 6.2 |
| HbA1c [mmol/mol] | 27 – 42 |
| TC [mmol/L] | 2.7 – 5.9 |
| LDL [mmol/L] | 1.3 – 4.0 |
| HDL [mmol/L] | 0.7 – 1.9 |
| TG [mmol/mol] | 0.42 – 2.8 |
| ALT [mikrokat/L] | < 0.5 |
| AST [mikrokat/L] | < 0.78 |
|  |  |
